# Alterations in airway mechanics, inflammatory biomarkers and lung histopathology in a rat model of allergen-induced airway inflammation

**DOI:** 10.1186/1476-9255-10-S1-P8

**Published:** 2013-08-14

**Authors:** Stephen M Jordan, Rachel A Armstrong, Jennifer Hincks, David Bell, Kenneth G Meecham

**Affiliations:** 1 Department of Pharmacology, Huntingdon Life Sciences, Cambridgeshire, UK; 2 Department of Biomarkers, Bioanalysis and Clinical Sciences, Huntingdon Life Sciences, Cambridgeshire, UK; 3 Department of Pathology, Huntingdon Life Sciences, Cambridgeshire, UK

## 

The most objective indicator of asthma severity in the clinic is the measurement of reversible airway obstruction by spirometry. We evaluated the effect of antigen challenge on FEV_100_, FVC and PEF and airway cell infiltrate, cytokine levels and lung histopathology in the Brown Norway rat allergic model. Rats were sensitised to the antigen ovalbumin and challenged by aerosolised ovalbumin fourteen days later. Twenty four hours after challenge the rats were terminally anaesthetised and a forced manoeuvres procedure performed. Recruitment of inflammatory cells and biomarker production was assessed in bronchoalveolar lavage fluid (BALF). Antigen challenge caused a significant (P<0.001) 30.2±2.6, 20.5±1.7 and 43.0±3.2% reduction in FEV_100_, PEF and FVC.

Exposure to antigen also resulted in the significant recruitment of eosinophils (2.13±0.60x10^6^cells/animal), neutrophils (2.64±36x10^6^cells/animal) and lymphocytes (0.53±0.05x10^6^cells/animal) into the airway. The Th2 cytokines; IL-13 and IL-5 and macrophage derived TNF-α in addition to IL-6 and MIP-1α levels were significantly elevated in the BALF. Oral treatment with a glucocorticoid steroid budesonide (3 mg/kg) twice daily completely reversed the decline in FEV_100_, PEF and FVC and significantly (P<0.001) reduced the inflammatory cell infiltrate, cytokine secretion and reduced the percentage incidences and severity of granulomatous inflammation. We have demonstrated that allergen challenge results in a reversible decline in measured FEV_100_, PEV and FVC in a rat allergic model. This functional measurement may be a valuable tool for translating the efficacy of novel compounds from rodents to the clinic.

